# Rosai–Dorfman Disease: Breast Involvement—Case Report and Literature Review

**DOI:** 10.3390/medicina57111167

**Published:** 2021-10-27

**Authors:** George Iancu, Nicolae Gica, Laura Mihaela Mustata, Anca Maria Panaitescu, Danut Vasile, Gheorghe Peltecu

**Affiliations:** 1 Department of Obstetrics and Gynecology, Filantropia Clinical Hospital of Obstetrics and Gynecology, 11-13 Ion Mihalache Bvd, 011171 Bucharest, Romania; george.iancu@umfcd.ro (G.I.); lauramustata@yahoo.com (L.M.M.); anca.panaitescu@umfcd.ro (A.M.P.); gheorghe.peltecu@umfcd.ro (G.P.); 2 Department of General Surgery, Emergency University Hospital, 169 Independentei Street, 050098 Bucharest, Romania; danutvasiledr@yahoo.com; 3 Department of Obstetrics and Gynecology, Faculty of Medicine, “Carol Davila” University of Medicine and Pharmacy, 37 Dionisie Lupu Street, 020021 Bucharest, Romania

**Keywords:** Rosai–Dorfman disease, Rosai–Dorfman of breast, breast pathology, histiocytosis

## Abstract

*Background and objectives*: Rosai–Dorfman disease (RDD) is a type of histiocytosis that usually appears in young adults or children as bilateral cervical lymphadenopathy, but extranodal involvement in not uncommon. Although the pathogenesis is not entirely elucidated, recent studies showed a possible neoplastic process. *Materials and methods*: Our manuscript presents a rare case of Rosai–Dorfman disease of the breast, the management of this rare case, and a literature review. There are few cases reported of RDD of the breast (around 90 globally reported cases); the data is poor, and the management not yet standardized for these cases. The case reported here shows the importance of correct breast investigation, breast imaging, and ultrasound-guided biopsy that provided an accurate diagnosis and guided further management. *Results*: Although RDD of the breast was rarely presented as bilateral disease in other case reports, our case showed bilateral breast disease with the suspicion of breast cancer on imaging. Pathology and immunohistochemistry were of critical importance and showed a specific pattern for histiocytosis. A multidisciplinary approach was taken into consideration for these cases in order to establish the approach. Some patients underwent surgery, but watchful waiting and close follow-up were the preferred approach. *Conclusions*: RDD of the breast is a rare form of histiocytosis, with fewer than 100 globally published cases. Although the management of this disease is not established yet by guidelines, a follow-up approach should be enough for these patients, and surgery might be overtreatment. Mortality from RDD is very low due to comorbidities. A multidisciplinary team decision is important, and abstinence might significantly benefit these patients.

## 1. Introduction

Rosai–Dorfman disease (RDD) is a histiocytosis that involves usually cervical lymph nodes in children and young adults. However, extranodal involvement is not uncommon, often raising diagnostic and therapeutic difficulties. It was described by a French pathologist, Pierre Destombes, as early as 1965, and was then thought to be a lipid storage disease occurring as a consequence of inflammation [1]; four years later, Rosai and Dorfman correctly identified the key roles of histiocytes in the pathogeny of disease, most often presenting as bilateral cervical painless lymphadenopathies, and communicated 34 similar cases with voluminous lymphadenopathy [2,3,4].

Currently, it belongs to R-group histiocytosis according to the revised classification of The Histiocyte Society, which includes familial RDD, classical (nodal) RDD, extranodal RDD, neoplasia-associated RDD, immune disease-associated RDD, and other miscellaneous types of histiocytosis [5].

Extranodal RDD is encountered in over 40% of patients and usually occurs concomitant with nodal disease. The most frequently involved extranodal localizations cited in the literature are the nasal cavity, skin, orbits, bone and central nervous system, but also skeletal muscle, subcutaneous tissue, heart, or thyroid. Breast localization is rare and often causes difficulties in differential diagnosis with breast malignancies. Other localizations are also cited, such as in the eyes, gastrointestinal tract, or uterine cervix [6,7].

The etiology of the disease is still unknown, and its natural history is marked by long phases of remission and relapse. Some theories suggests immune regulation disorder or infection etiology such as the varicella zoster virus, herpes virus, Epstein–Barr virus, cytomegalovirus or HIV [8,9].

Clinically, the classical nodal disease is painless, and examination reveals bilateral cervical lymphadenopathy in most cases. Associated symptoms can be fever and night sweats or weight loss. Apart from cervical lymphadenopathy, other groups of lymph nodes can also be involved (axillary, inguinal, mediastinal, or rarely retroperitoneal) [10,11].

Apart from the classical nodal RDD, extranodal RDD can involve a large variety of localization, with symptoms according to the involved organ (double vision or orbital pain, nasal obstruction or epistaxis, oral pain, dyspnea, cough, abdominal pain, hematochezia, hematuria, bone pain, skin changes, headaches, seizures, or other neurologic manifestations) [12].

RDD on laboratory evaluation is associated with a large number of neutrophil cells on full blood count with differential polyclonal hypergammaglobulinemia on serum immunoglobulins testing and high erythrocyte sedimentation rate. Other laboratory tests to be considered for differential diagnosis or associated disease identification are antinuclear antibodies, HLA-B27, or autoimmune lymphoproliferative syndrome markers, complete metabolic panel, blood smear or bone marrow aspirate, and lumbar puncture [12].

Pathology examination for nodal RDD reveals large histiocytes with pale cytoplasm and large hypochromatic nucleus and sinus expansion. Immunohistochemistry shows nuclear and cytoplasmic S-100 and CD68 positivity, and sometimes CD163 and CD14 positivity. RDD is differentiated by Langerhans cell histicytosis by CD1a and CD207 negativity. These characteristics help differentiate RDD from Langerhans cell histiocytosis and Erdheim–Chester disease. Emperipolesis (intact leukocytes in the cytoplasm of histiocytes) is a useful marker but is not a mandatory finding for diagnosis, as it is sometimes missed on evaluation because of the focal presence, usually in extranodal sites, and it can also be found in other pathologies, namely, Erdheim–Chester disease, juvenile xanthogranuloma, or malignant histiocytosis [12].

Lymphoma and infectious diseases such as tuberculosis are the most common clinical entities that can mimic RDD, especially in developing countries. Moreover, the disease appears more often in male children or young adults. The unusual presentation of breast lumps in premenopausal women can easily be considered to be a breast malignancy, this being atypical for RDD [13]. 

The treatment strategies for RDD include an expectative approach and monitoring, corticosteroids (usually prednisone or dexamethasone), surgery for unifocal and/or symptomatic extranodal disease, radiotherapy (with a dose of 30–50 Gy), chemotherapy (Vinca alkaloids, methotrexate, cladribine), or immunomodulatory therapy (thalidomide, lenalidomide, rituximab, imatinib mesylate) [12,14,15].

Although the clinical course is unpredictable and marked by long phases of remission with periods of relapse and worsening symptoms, the disease seems to be self-limiting in many cases in which observation is reasonable, wiith 20%–50% of patients with nodal or cutaneous RDD experiencing spontaneous remission. However, evidence is scarce because of the lack of randomized trials. The overall outcome and prognosis of RDD are usually good, and treatment is necessary in selected cases with important clinical signs such as vital-organ compression and airway obstruction due to the impressive node enlargement [15].

Outcome is favorable in the majority of cases, especially for nodal or cutaneous diseases; death as a direct result of RDD is rare, cited between 7% in one of the largest series of patients with RDD published and 12% in a comprehensive review published in 2002 [15,16].

We analyzed extranodal RDD involving the breast in our review because it is a rare condition, currently described in the literature in only about 90 cases, with non-standardized management because of the rarity of the disease and because existing evidence regarding the management of this localization is still scarce.

## 2. Objective

The principal objective of this comprehensive review and case report is to bring new insights and to systematize the existing data in the literature about this rare localization of RDD, helping to increase the accuracy of diagnosis and adequacy of treatment of this rare condition of the breast.

## 3. Case Presentation

We present the case of a 63-year-old woman with a personal history of renal neoplasia (transitional cell carcinoma surgically treated eight years ago, free of disease at present) and systemic histiocytosis (bone located), and a family history of lung carcinoma (father) and thyroid carcinoma (daughter). Systemic histiocytosis was diagnosed six years ago on a surgical specimen from a tibial bone tumor. No pathology results were available. Thereafter, she did not have any other manifestations of disease until breast lumps were diagnosed on screening mammogram. She was not under any treatment for her systemic histiocytosis. 

She presented with bilateral breast lumps for investigation and management. Clinical examination revealed bilateral breast lumps on the right side in the axillary tail of the right breast, a palpable lump of about 2.5 cm with irregular borders, not infiltrating the skin or muscles, and on the left side, at the border of inferior quadrants, a 1.5 cm lump with similar characteristics; no palpable suspicious axillary lymph nodes were clinically identified. No other suspicious lesions located outside the breasts were identified.

**Figure 1:**
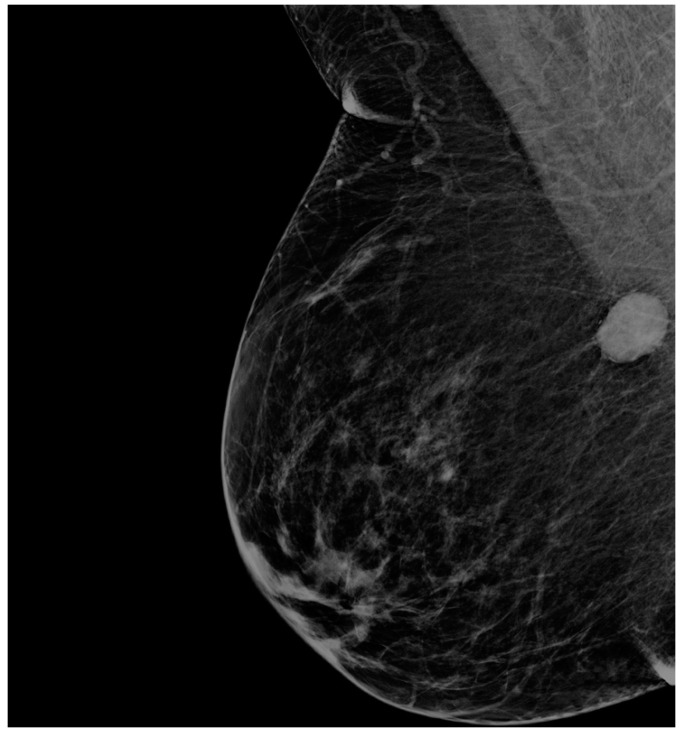
Right breast mammogram—mediolateral oblique incidence.

**Figure 2:**
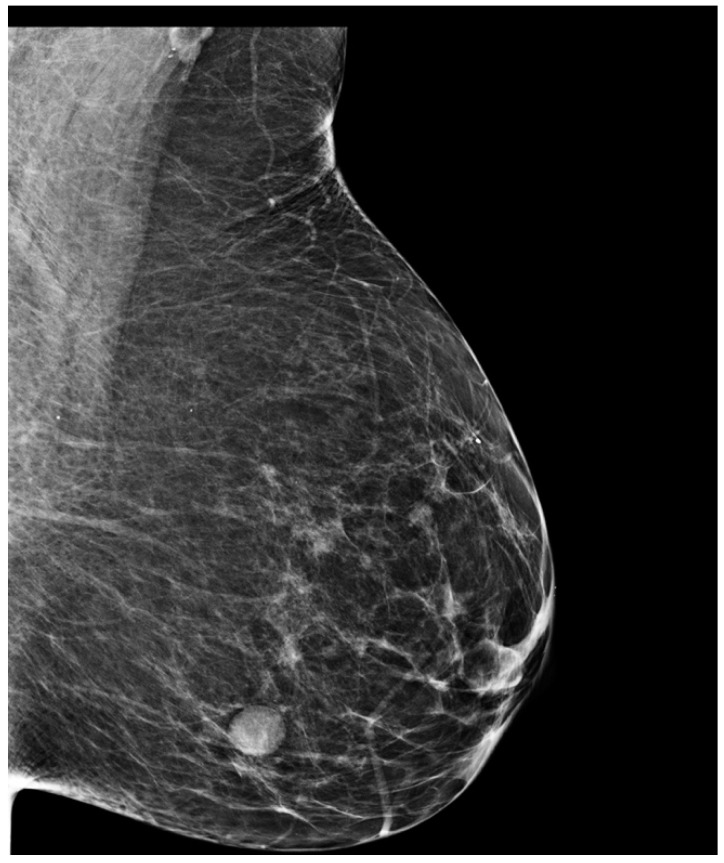
Left breast mammogram—mediolateral oblique incidence.

Mammography revealed bilateral suspicious findings: an opacity on the right side towards axillary tail with diameters 2.0/1.6 cm, slightly irregular, and on the left side a similar image of 1.3/1.4 cm, distributed at the border between inferior quadrants (Figure 1 and Figure 2). Ultrasound imaging confirmed the two above-described lesions. They were both scored BIRADS 4a. There were no suspicious axillary lymph nodes visualized on imaging.

Bilateral ultrasound-guided 14-gauge core biopsies of the breast lumps were undertaken. Pathology exam findings (haematoxylin–eosin and immunohistochemistry) revealed histiocyte aggregates, emperipolesis, histiocytes diffuse positive for S100 protein, CD1a, focally expressing CD68, negative for CD20 and CD3. Pathology findings were consistent with the diagnosis of bilateral Rosai–Dorfman disease of the breast. 

**Figure 3:**
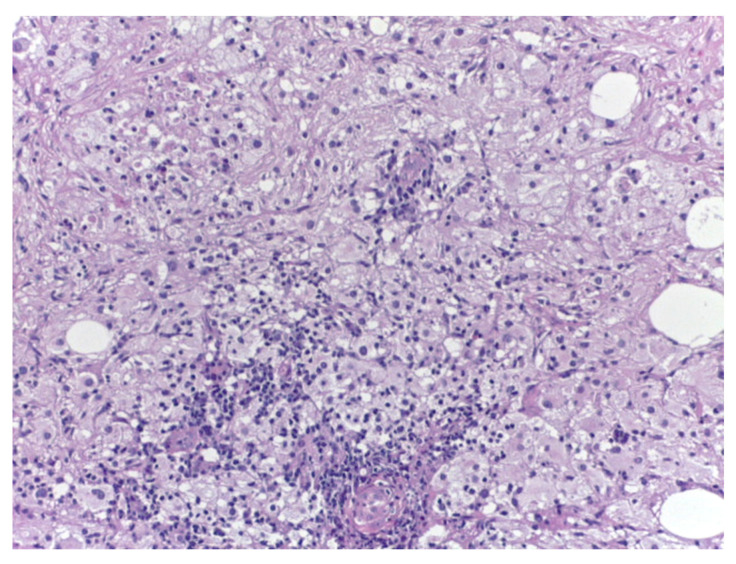
Rosai–Dorfman disease of the breast—haematoxylin–eosin (20×).

**Figure 4:**
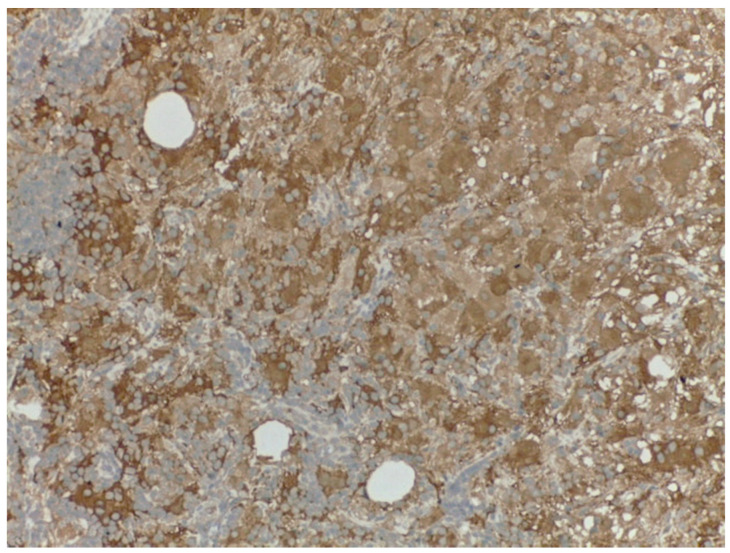
Rosai–Dorfman disease of the breast: immunohistochemistry—S100 diffuse positive histiocytes (20×).

**Figure 5:**
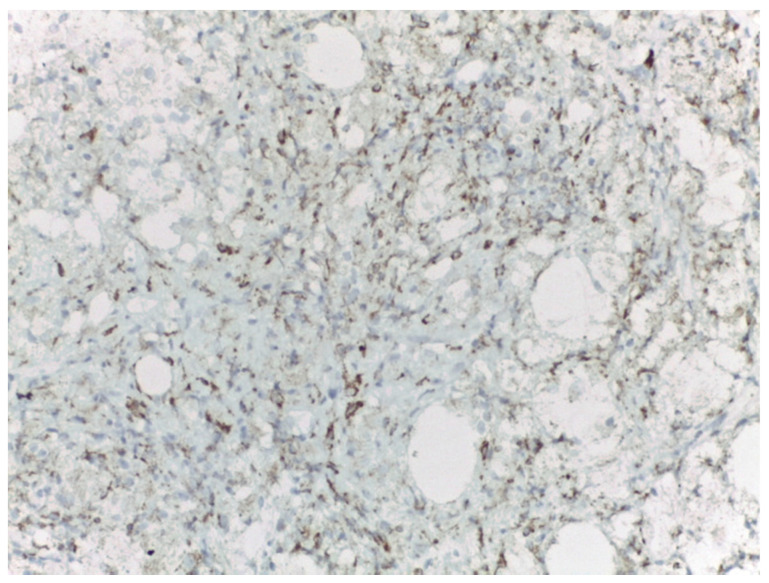
Rosai–Dorfman disease of the breast: immunohistochemistry—CD68 focally positive histiocytes (20×).

**Figure 6:**
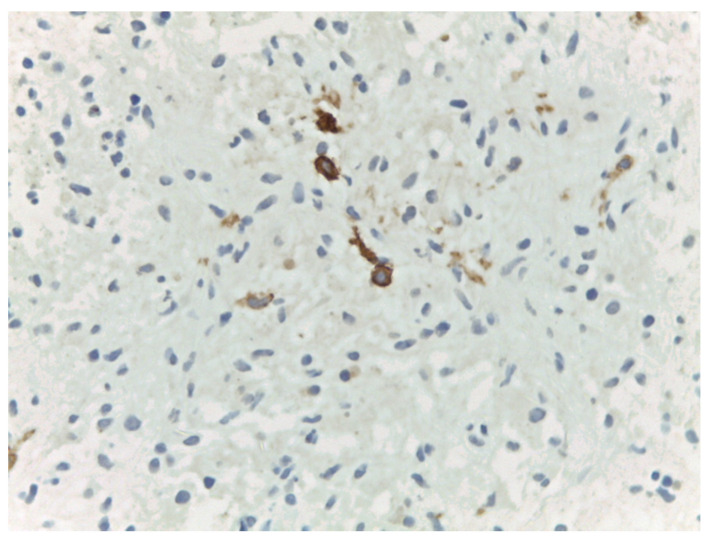
Rosai–Dorfman disease of the breast: immunohistochemistry—CD1a positive histiocytes (40×).

Differential diagnosis with benign breast pathology and with invasive mammary carcinoma was performed. Pathology findings of emperipolesis and positive staining for S100 helped in the final positive diagnosis of extranodal RDD of the breast (Figure 3, Figure 4, Figure 5 and Figure 6).

The multidisciplinary team meeting decision was to continue surveillance with imaging in the absence of any medical or surgical treatment. 

The patient repeated ultrasound assessment at 6 months and mammogram at one year follow-up. The breast lumps disappeared at 6-month follow-up without any treatment.

The particularity of our case is the early diagnosis after suspicious imaging using an ultrasound-guided core biopsy in a patient with a suggestive personal history of RDD and conservative management of the breast tumour with follow-up imaging, avoiding unnecessary surgery. 

## 4. Materials and Methods

For the present comprehensive review, we selected all published original articles communicating confirmed cases on pathology with extranodal Rosai–Dorfman disease located in the breast and/or axilla. 

A comprehensive review of the literature was undertaken; eligibility criteria circumscribed all original articles reporting on extranodal Rosai–Dorfman disease of the breast published in the literature, including case reports and case series. All selected papers’ reported cases confirmed on pathology with breast and/or axillary localization. 

**Figure 7:**
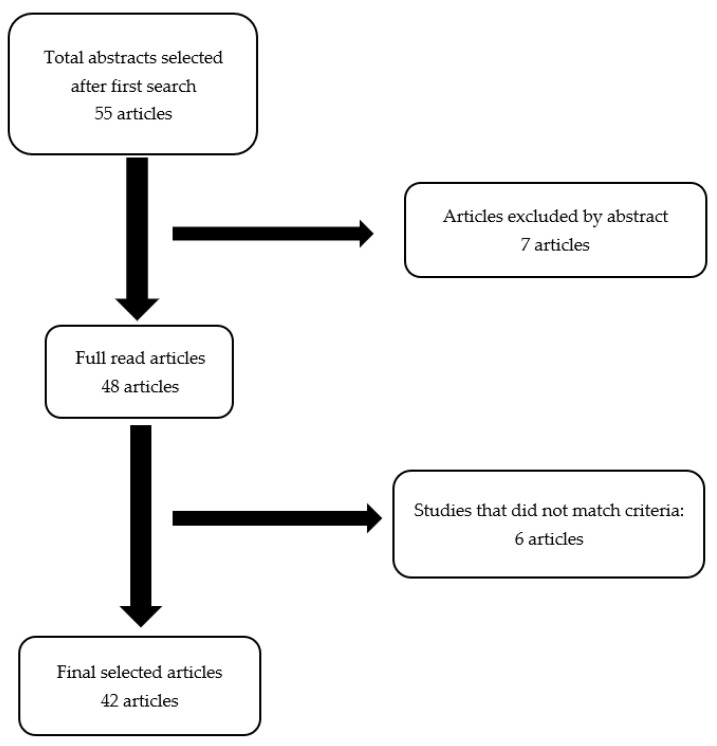
Algorithm of article selection.

A literature search was undertaken using as keywords “Rosai–Dorfman disease”, “histiocytosis”, and “breast”. The PubMed, Embase, and Scopus databases were searched. Articles published in English until April 2021 were selected, although other languages were not excluded (Spanish, Dutch, Chinese). We found 55 articles; of these, 7 articles were excluded after reading the abstract, and 6 articles after reading the full text. The excluded articles were not relevant to our study because they were review papers or lacking original case reports, or RDD was not located in the breast. We selected 42 articles after reading the abstract and full text (selection algorithm in Figure 7). Selected articles are systematized in Table 1 [17,18,19,20,21,22,23,24,25,26,27,28,29,30,31,32,33,34,35,36,37,38,39,40,41,42,43,44,45,46,47,48,49,50,51,52,53,54,55,56,57,58]. The strength of evidence-based data was low because of the rarity of RDD localization and the small number of patients; only case reports, case series, and retrospective trials were found on extensive search, and no prospective or randomized data were identified.

## 5. Results

A total of 92 patients with a diagnosis of Rosai–Dorfman disease of the breast on pathology were selected and analysed. Most literature communications were case reports or case series. The median aged was 55 years old (15–84), while gender distribution was dominated by females (10 males to 82 females).

Imaging was performed in most patients, usually using ultrasound and/or mammogram, often followed by imaging-guided core biopsy. Axillary involvement was described in 5/92 patients (5.4%), which confused the clinical picture even more, orientating the clinical suspicion towards malignancy.

Multicentric appearance at diagnosis was rather rare, communicated in 7 out of 78 patients (9.0%). Bilateral breast involvement with data provided in the selected papers was described in 5/78 patients for (6.4%). Recurrent disease was uncommon, cited in 6/64 patients (9.4%) (for28 patients, there was no follow-up reported). There was no significant association between axillary or bilateral breast involvement and systemic disease manifestations.

Pathology diagnosis is critically important in breast abnormalities. New pathology categories based on immunohistochemistry are currently researched [59]. RDD of the breast was definitively diagnosed on pathology either on biopsy or on surgical specimen. Of the patients, 55/92 (59.8%) underwent core-needle biopsy, while the rest had a diagnosis on the surgical specimen. Pathology diagnosis was conducted by the identification of large histiocytes with a round nucleus containing vesicular chromatin with a couple of nucleoli and pale cytoplasm; the typical finding was emperipolesis (from Greek: em—inside, peri—around, polemai—wander about), a phenomenon consisting of the presence of lymphocytes in the cytoplasm of other cells, usually histiocytes; it is defined as the penetration of one living cell by another cell that remains intact, distinct from phagocytosis. [60]

Pathology diagnosis was eased by immunohistochemistry staining of S-100 protein and CD-68 in most cases, characteristic for antigen-presenting or Langerhans cells. Most presented cases of RDD of the breast did not have molecular study evaluation or assessed BRAF mutations.

More than half of the patients (56 out of 92 patients—60.9%) underwent surgery, usually lumpectomy or even mastectomy [20]. The rest were approached expectantly; steroids were administered in a minority of patients.

Follow-up information varied from lost to follow-up to 3–12 months, while a few studies cited 2 or 3 years follow-up [42,44]. The course of the breast RD disease was usually uneventful with spontaneous remission in the majority of patients and with no recurrence in patients that underwent surgery. Breast recurrence was usually the exception during the course of disease [24,30], while fatalities during follow-ups were also very rare and due to other causes (acute leukemia, respiratory failure [23]).

## 6. Discussion

Our study sheds more light in the rare pathology of RDD of the breast and systematises existing data in the literature regarding the diagnosis and management of this condition. Because of the paucity of literature data, the differential diagnosis and management of RDD of the breast are still debated, with the current data reflecting the difficulty of differential diagnosis and frequent surgical overtreatment.

Breast involvement in Rosai–Dorfman disease is a rare occurrence, but it can be mistakenly considered to be a malignant condition on clinical examination, imaging, or even pathology [30]. The involvement of the axilla can induce even more confusion for the clinician. Most cases communicated in the literature were postmenopausal women diagnosed after 50 years of age using common breast imaging tools such as ultrasound and mammogram. A personal history of Rosai–Dorfman disease helped in raising suspicions of extranodal RDD. Final diagnosis was performed on pathology on either core needle biopsy or excisional specimen. Emperipolesis on HE staining and staining for CD-68 and S-100 on immunohistochemistry were cardinal features for diagnosis.

Differential diagnosis on pathology is usually performed with inflammatory myofibroblastic tumours, granulomatous mastitis, IgG4-sclerosing mastitis, or breast lymphoma with plasmocytic differentiation.

In our patient, the diagnosis of RDD of breast was eased by the personal history of histiocytosis, although imaging was suspicious of malignancy. Core needle biopsy limited the morbidity of an excisional biopsy and pathology diagnosis of RDD of the breast spared the patient any surgical treatment, leading to spontaneous disappearance of bilateral breast nodules at one year follow-up.

Treatment of this localization of RDD was not necessary in most of the cases. However, due to paucity of literature data, many patients underwent surgical excision or lumpectomy, with diagnostic and curative intent. Only one case of radical surgery (mastectomy) was found in the literature [20].

The outcome of RDD breast localization seems to be very good even without any intervention. The occasional progression or recurrence of the disease was cited in the literature, and mortality was exceptionally communicated, usually due to other comorbidities.

## 7. Conclusions

RDD of the breast is a rare localization that can confound diagnosis and lead to surgical overtreatment. Clinicians should be aware of its existence and raise suspicion, especially in patients with personal history of RDD. Core needle biopsy is needed for definitive diagnosis; clinicians should effectively communicate with pathologists for a timely and correct diagnosis. Pathology diagnosis of emperipolesis and positive staining on immunohistochemistry for S100 protein and CD-68 is of utmost importance. Until more evidence-based data are available, a conservative approach with abstention from any kind of surgery and clinical follow-up would spare the patients the risks of unnecessary treatment.

## Figures and Tables

**Table 1:** Selected articles and patient characteristics.

Author	No of pts	Age	Breast Side	Breast Localization	Axillary	Diagnosis	Pathology	Mangement	FUp	Gender	Observations
Battle 2021 [17]	1	49	RB	Multifocal, UIQ	no	1,2,3	1	1	N/A	F	N/A
Delaney 2017 [18]	1	63	RB	UOQ	no	1,2,3	1,2	1	1	F	N/A
Liu 2018 [19]	4	46–68	N/A	N/A	no	1,2,3	1	1	N/A	F	N/A
Hoffmann 2019 [20]	22	median 54 (range 37–71)	RB, LB	N/A	no	1,2,3	1,2	4 cases—1; 18 cases—2; (1 case mastect)	4 cases—1; 2 cases—2	18—F, 4—M	N/A
Chen 2016 [21]	12	median 37 (range 28–47)	7—RB, 5—LB	N/A	yes—1 no—11	1,2,3	2	N/A	N/A	12—F	Article in chinese
El-Attrache 2018 [22]	1	55	RB	LQ	no	1,3	1,2	2	2	F	Breast recurrence of RDD
Shetty 2020 [23]	4	median 58 (range 43–69)	N/A	N/A	yes—2 no—2	3—1,2	1,2	2	3 cases—1; 2 cases—3 1 case—N/A	F	N/A
Shin 2020 [24]	1	54	RB	OUQ	no	1,2,3	1,2	2	2	F	N/A
de Mello Tucundova 2017 [25]	1	55	LB	N/A	no	1,2,3	1,2	2	N/A	F	N/A
Jorns 2017 [26]	1	N/A	LB	N/A	no	1,2,3	1,2	1	1	F	No recurrence
Goldbach 2019 [27]	1	44	RB	UIQ	no	1,2,3	1,2	2	1	F	Recurrence at 6 months
Parkin 2015 [28]	1	56	LB	N/A	no	1,2,3	1,2	1	1	F	N/A
Ciurea 2016 [29]	1	59	RB	UIQ	no	1,2,3	2	1	1	F	Hyperpigmentation of tegument
Tenny 2011 [30]	1	64	RB	N/A	yes	1,2,3	1,2	2	2	F	Multiple distant recurrence at 6 months
Morkowski 2010 [31]	3	median 51 (range 39–62)	2 LB, 1 RB	2 UOQ 1 UIQ	no	1,2,3	1,2	2	1	F	No recurrence after excision
Vaidya 2020 [32]	1	43	LB	UOQ	no	1,2,3	1,2	2	1	F	N/A
Simmons 2016 [33]	1	41	RB	Multifocal	no	1,2,3	1,2	2	1	F	No recurrence at 9 months FU
Zhou 2016 [34]	1	71	LB	Multifocal	no	1,2,3	1,2	2	1	F	N/A
Green 1997 [35]	7	median 46 (range 15–84)	4 RB, 2 LB 1 axillary	2—bilateral	yes—1 no—6	1,2,3	1,2	6—2 1—1	N/A	F	N/A
Cha 2012 [36]	1	62	RB	LOQ	no	1,2,3	1,2	2	1	F	No recurrence at 10 months FU
da Silva 2007 [37]	1	50	LB	UOQ	no	1,2,3	1,2	2	1	F	N/A
Ng 2000 [38]	2	median 50 (47–58)	RB	UOQ	no	1,FNA 1,2	1,2	2	1	F	No recureence at 1 and 6 months FU
Moyon 2020 [39]	1	29	N/A	N/A	no	1,2,3	1,2	1	N/A	F	N/A
Bansal 2010 [40]	1	35	RB	LOQ	no	1, FNA	1,2	1	1	M	No recurrence at 18 Mo FU
Fu 2012 [41]	1	78	RB	UOQ	no	PET/CT scan	1,2	2	N/A	F	N/A
Mantilla 2016 [42]	2	63	LB	1 multicentric	no	1,2,3	1,2	1	1	F	At 3 years FU—subcutaneous soft mass
Krbanevic 2021 [43]	1	50	Bilateral	N/A	no	1,3	1	1	N/A	F	N/A
Wu YC 2010 [44]	1	33	RB	UOQ	no	1,3	2	2,3	1	F	No recurrence at 2 years FU
Mac-Moune Lai 1994 [45]	1	34	LB	LIQ	no	1,2	2	2	1	M	No recurrence at 3 months FU
Wang 1997 [46]	1	35	LB	N/A	no	1,2,3	2	2	2	F	Recurrent breast tumor
Gwin 2011 [47]	1	68	Bilateral	N/A	no	1,2,3	1,2	1	N/A	F	N/A
Noordzij 2011 [48]	1	75	RB	Multifocal	no	1,3	1,2	1	N/A	F	Article in Dutch
Pham 2005 [49]	1	53	LB	LIQ	no	1,2,3	1,2	1	N/A	F	N/A
Elshikh 2020 [50]	3	60	LB	N/A	no	1,2	1,2	1	N/A	F	N/A
Hammond 1996 [51]	1	67	RB	UOQ	no	2,3	1	2	1	F	No recurrence at 6 Mo FU
Kuzmiak 2003 [52]	1	30	RB	UOQ, Multifocal	no	1,2,3	1,2	2	N/A	F	N/A
Baladandapani 2012 [53]	1	59	LB	Multifocal, UQ	no	1,2,3	1,2	2	N/A	M	N/A
Hummel 1999 [54]	1	52	LB	UIQ	no	1	1,2	2	1	F	No recurrence at 11 Mo FU
Dahlgren 2008 [55]	1	64	Bilateral	Bilateral	no	1,2,3	1,2,	2	N/A	F	N/A
Dias Perera 2007 [56]	1	23	LB	N/A	no	1,2	2	2	N/A	M	N/A
Picon-Coronel 2010 [57]	1	67	N/A	N/A	no	1,2	1,2	2	N/A	F	Aticle in spanish
Perez-Guillermo 1993 [58]	1	71	Bilateral	UIQ left, LIQ right	no	1,2	1,2	2	N/A	F	N/A

Abbreviations: F- Female, M- Male, LB—left breast, RB—right breast, N/A not available, FU—follow up, UOQ—upper outer quadrant, UQ—upper quadrants, UIQ—upper inner quadrant, LOQ—lower outer quadrant, LIQ—lower inner quadrant; FNA—fine needle aspiration. Diagnosis—1—US; 2—Mammogram; 3—Biopsy. Pathology—1—emperipolesis, 2—S100 positive, 3—CD46 positive. Management—1—expectant, 2—surgery, 3—steroids. Follow-up—FU—1—no progress or recurrence, 2—recurrence, 3—death.

## Data Availability

The PubMed, Embase, and Scopus databases were searched. Articles published in English until April 2021.

## References

[1] Destombes P. (1965). Adenitis with lipid excess, in children or young adults, seen in the Antilles and in Mali (4 cases). Bull. Soc. Pathol. Exot..

[2] Rosai J., Dorfman R.F. (1969). Sinus histiocytosis with massive lymphadenopathy. A newly recognized benign clinicopathological entity. Arch. Pathol..

[3] Symss N., Cugati G., Vasudevan M., Ramamurthi R., Pande A. (2010). Intracranial Rosai Dorfman Disease: Report of three cases and literature review. Asian J. Neurosurg..

[4] Rosai J., Dorfman R.F. (1972). Sinus histiocytosis lymphadenopathy: A pseudolymphomatous disease: Analysis of 34 cases. Cancer.

[5] Emile J.F., Abla O., Fraitag S., Horne A., Haroche J., Donadieu J., Requena-Caballero L., Jordan M.B., Abdel-Wahab O., Allen C.E. (2016). Histiocyte Society. Revised classification of histiocytoses and neoplasms of the macrophage-dendritic cell lineages. Blood.

[6] Bruce-Brand C., Schneider J.W., Schubert P. (2020). Rosai-Dorfman disease: An overview. J. Clin. Pathol..

[7] Sharma M.S., De Padua M., Jha A.N. (2005). Rosai-Dorfman disease mimicking a sphenoid wing meningioma. Neurol. India.

[8] Paulli M., Bergamaschi G., Tonon L., Viglio A., Rosso R., Fachetti F., Geerts M.L., Magrini U., Cazzola M. (1995). Evidence for a polycloncal nature of the cell infiltrate in sinus histiocytosis with massive lymphadenopathy (Rosai Dorfman Disease). Br. J. Haematol..

[9] Delacrétaz F., Meugé-Moraw C., Anwar D., Borisch B., Chave J.P. (1991). Sinus histiocytosis with massive lymphadenopathy (Rosai Dorfman disease) in an HIV-positive patient. Virchows Archiv A Pathol. Anat. Histopathol..

[10] Al-Khateeb T.H. (2016). Cutaneous Rosai-Dorfman disease of the face: A comprehensive literature review and case report. J. Oral Maxillofac. Surg..

[11] Sodhi K.S., Suri S., Nijhawan R., Kang M., Gautam V. (2005). Rosai-Dorfman disease: Unusual cause of diffuse and massive retroperitoneal lymphadenopathy. Br. J. Radiol..

[12] Abla O., Jacobsen E., Picarsic J., Krenova Z., Jaffe R., Emile J.F., Durham B.H., Braier J., Charlotte F., Donadieu J. (2018). Consensus recommendations for the diagnosis and clinical management of Rosai-Dorfman-Destombes disease. Blood.

[13] Parkash O., Yousaf M.S., Fareed G. (2019). Rosai-Dorfman disease, an uncommon cause of common clinical presentation. J. Pak. Med. Assoc..

[14] Mohammadi O., Lisigurski M.Z., Mehra D., Pishdad R., Gulec S. (2020). Rosai-Dorfman Disease and Unusual Local Invasive Presentation. Cureus.

[15] Pulsoni A., Anghel G., Falcucci P., Matera R., Pescarmona E., Ribersani M., Villiva’ N., Mandelli F. (2002). Treatment of sinus histiocytosis with massive lymphadenopathy (Rosai-Dorfman disease): Report of a case and literature review. Am. J. Hematol..

[16] Foucar E., Rosai J., Dorfman R. (1990). Sinus histiocytosis with massive lymphadenopathy (Rosai-Dorfman disease): Review of the entity. Semin. Diagn. Pathol..

[17] Battle B., McIntire P., Babagbemi K., Mema E. (2021). Extranodal multifocal Rosai-Dorfman disease of the breast: A case report. Clin. Imaging.

[18] Delaney E.E., Larkin A., MacMaster S., Sakhdari A., DeBenedectis C.M. (2017). Rosai-Dorfman disease of the breast. Cureus.

[19] Liu M., Li X., Li Y., Wang Z., Cheng L., Song X., Wu Y. (2018). Rosai-Dorfman disease with features of IgG4-related disease in the breast: Cases report and literature review. Asian Pac. J. Allergy Immunol..

[20] Hoffmann J.C., Lin C.Y., Bhattacharyya S., Weinberg O.K., Chisholm K.M., Bayerl M., Cascio M., Venkataraman G., Allison K., Troxell M. (2019). Rosai-Dorfman disease of the breast with variable IgG4+ plasma cells: A diagnostic Mimicker of other malignant and reactive entities. Am. J. Surg. Pathol..

[21] Chen Y.P., Jiang X.N., Lu J.P., Zhang H., Li X.Q., Chen G. (2016). Clinicopathologic analysis of extranodal Rosai-Dorfman disease of breast: A report of 12 cases. Zhonghua Bing Li Xue Za Zhi Chin. J. Pathol..

[22] El-Attrache B., Gluck B., Heimann A., Kapenhas E. (2018). A rarity in breast pathology: First recurrent male case of Rosai-Dorfman disease. Int. J. Surg. Case Rep..

[23] Shetty S., Sharma N., Booth C.N., Oshilaja O., Downs-Kelly E.P., McKenney J.K., Sturgis C.D. (2020). Mammary extranodal Rosai-Dorfman disease with and without associated axillary lymphadenopathy: Insights for practitioners of breast pathology. Int. J. Surg. Pathol..

[24] Shin G.W., Park Y.M., Heo Y.J., Baek J.W., Lee Y.J., Han J.Y., Park H. (2020). Sonographic features of Rosai-Dorfman disease in the breast: A case report. J. Clin. Ultrasound.

[25] De Mello Tucunduva T.C., Gaziero A., Tostes V.S., Chaves M.C., Stiepcich M.M.A., Torres U.S., Chala L.F., de Mello G.G.N. (2019). Extranodal Rosai-Dorfman disease manifesting with breast involvement: Imaging and histopathological findings. Breast J..

[26] Jorns J.M. (2017). Extranodal Rosai-Dorfman Disease of the Breast. Breast J..

[27] Goldbach A.R., Hava S., Caroline D., Zhao X., Bains A., Pascarella S. (2019). Rosai-Dorfman disease of the breast: A potential marker of systemic disease. Breast J..

[28] Parkin C.K.E., Keevil C., Howe M., Maxwell A.J. (2015). Rosai-Dorfman disease of the breast. BJR Case Rep..

[29] Ciurea A., Ciortea C., Cosarca M., Rogojan L. (2016). Breast Involvement in Pure Cutaneous Rosai-Dorfman Disease: Ultrasound and Sonoelastography appearance with a review of the literature. Ultrasound Q..

[30] Tenny S.O., McGinness M., Zhang D., Damjanov I., Fan F. (2011). Rosai-Dorfman Disease Presenting as a Breast Mass and Enlarged Axillary Lymph Node Mimicking Malignancy: A Case Report and Review of the Literature. Breast J..

[31] Morkowski J.J., Nguyen C.V., Lin P., Farr M., Abraham S.C., Gilcrease M.Z., Moran C.A., Wu Y. (2010). Rosai-Dorfman disease confined to the breast. Ann. Diagn. Pathol..

[32] Vaidya T., Mahajan A., Rane S. (2020). Multimodality imaging manifestations of Rosai-Dorfman disease. Acta Radiol. Open.

[33] Simmons N.R., Xu M.L., Tavassoli F.A., Geisel J., Killelea B., Philpotts L.E. (2016). A Rare Presentation of Rosai-Dorfman Disease as a Breast Mass. Breast J..

[34] Zhou Q., Ansari U., Keshav N., Davis F., Cundiff M. (2016). Extranodal manifestation of Rosai-Dorfman disease in the breast tissue. Radiol. Case Rep..

[35] Green I., Dorfman R.F., Rosai J. (1997). Breast involvement by extranodal Rosai-Dorfman disease: Report of seven cases. Am. J. Surg. Pathol..

[36] Cha Y.J., Yang W.I., Park S.H., Koo J.S. (2012). Rosai-Dorfman disease in the breast with increased IgG4 expressing plasma cells: A case report. Korean J. Pathol..

[37] Da Silva B.B., Lopes-Costa P.V., Pires C.G., Moura C.S., Borges R.S., da Silva R.G. (2007). Rosai-Dorfman disease of the breast mimicking cancer. Pathol. Res. Pract..

[38] Ng S.B., Tan L.H., Tan P.H. (2000). Rosai-Dorfman disease of the breast: A mimic of breast malignancy. Pathology.

[39] Moyon Q., Boussouar S., Maksud P., Emile J.F., Charlotte F., Aladjidi N., Prévot G., Donadieu J., Amoura Z., Grenier P. (2020). Lung involvement in Destombes-Rosai-Dorfman disease: Clinical and radiological features and response to the MEK inhibitor cobimetinib. Chest.

[40] Bansal P., Chakraborti S., Krishnanand G., Bansal R. (2010). Rosai-Dorfman disease of the breast in a male: A case report. Acta Cytol..

[41] Fu L., Liu M., Song Z., Xu B., Tian J. (2012). 18 F-fluoro-deoxyglucose positron emission tomography/computed tomography scan findings in Rosai-Dorfman disease with IgG4-positive plasma cell infiltration mimicking breast malignancy: A case report and literature review. J. Med. Case Rep..

[42] Mantilla J.G., Goldberg-Stein S., Wang Y. (2016). Extranodal Rosai-Dorfman disease: Clinicopathologic series of 10 patients with radiologic correlation and review of the literature. Am. J. Clin. Pathol..

[43] Krbanjevic A., Brown H.G. (2021). Rosai-Dorfman disease extending to the brain. Clin. Neuropathol..

[44] Wu Y.C., Wang C.H., Lin Y.Y., Yen K.Y., Hsieh T.C., Sun S.S., Chang H.W., Kao C.H. (2010). A mimic of breast lymphoma: Extranodal Rosai-Dorfman disease. Am. J. Med. Sci..

[45] Mac-Moune Lai F., Lam W.Y., Chin C.W., Ng W.L. (1994). Cutaneous Rosai-Dorfman disease presenting as a suspicious breast mass. J. Cutan. Pathol..

[46] Wang J.S., Hsieh S.P., Shih D.F., Tseng H.H. (1997). Cutaneous Rosai-Dorfman disease manifestating as recurrent breast tumor: A case report. Zhonghua Yi Xue Za Zhi Chin. Med. J..

[47] Gwin K., Cipriani N., Zhang X., Schmidt R., Hyjek E. (2011). Bilateral Breast Involvement by Disseminated Extranodal Rosai-Dorfman Disease. Breast J..

[48] Noordzij W., Weernink E.E., van Imhoff G.W., Kluin P.M., de Haan L.D. (2011). Benign histiocytosis: Rosai-Dorfman disease. Ned. Tijdschr. Geneeskd..

[49] Pham C.B., Abruzzo L.V., Cook E., Whitman G.J., Stephens T.W. (2005). Rosai-Dorfman disease of the breast. Am. J. Roentgenol..

[50] Elshikh M., Schellingerhout D., Rayan J., Taher A., Elsayes A.K., Mujtaba B., Garg N. (2020). Disease characteristics, radiologic patterns, comorbid diseases, and ethnic differences in 32 patients with Rosai-Dorfman disease. J. Comput. Assist. Tomogr..

[51] Hammond L.A., Keh C., Rowlands D.C. (1996). Rosai-Dorfman disease in the breast. Histopathology.

[52] Kuzmiak C.M., Koomen M., Lininger R., Pisano E. (2003). Rosai-Dorfman disease presenting as a suspicious breast mass. Am. J. Roentgenol..

[53] Baladandapani P., Hu Y., Kapoor K., Merriam L., Fisher P.R. (2012). Rosai-Dorfman disease presenting as multiple breast masses in an otherwise asymptomatic male patient. Clin. Radiol..

[54] Hummel P., Waisman J., Chhieng D., Yan Z., Cohen J.M., Cangiarella J. (1999). Fine-needle aspiration cytology of Rosai-Dorfman disease of the breast: A case report. Diagn. Cytopathol..

[55] Dahlgren M., Smetherman D.H., Wang J., Corsetti R.L. (2008). Rosai-Dorfman disease of the breast and parotid gland. J. La. State Med. Soc. Off. Organ La. State Med. Soc..

[56] Dias Perera A.S., Keleher A.J., Nath M. (2007). Rosai-Dorfman disease presenting as a male breast mass. Am. Surg..

[57] Picón-Coronel G., Palmerín-Bucio M.E., Méndez-Pérez V., Alvarado-Cabrero I. (2010). Mammary gland Rosai Dorfman disease. A case report and literature review. Gac. Med. Mex..

[58] Pérez-Guillermo M., Sola-Perez J., Rodriguez-Bermejo M. (1993). Malacoplakia and Rosai-Dorfman disease: Two entities of histiocytic origin infrequently localized in the female breast—The cytologic aspect in aspirates obtained via fine-needle aspiration cytology. Diagn. Cytopathol..

[59] Iancu G., Vasile D., Iancu R.C., Davitoiu D.V. (2017). “Triple positive” breast cancer—A novel category. Rom. J. Morphol. Embryol..

[60] Rastogi V., Sharma R., Misra S.R., Yadav L., Sharma V. (2014). Emperipolesis—A review. J. Clin. Diagn. Res..

